# Challenges to conquer from the gender perspective in medicine: The case of spondyloarthritis

**DOI:** 10.1371/journal.pone.0205751

**Published:** 2018-10-12

**Authors:** Vega Jovani, Mar Blasco-Blasco, Eliseo Pascual, M. Teresa Ruiz-Cantero

**Affiliations:** 1 Rheumatology Department, Alicante University General Hospital, Alicante, Spain; 2 Public Health Department, University of Alicante, Alicante, Spain; 3 Department of Medicine, Miguel Hernandez University, Alicante, Spain; 4 CIBERESP (Centro de Investigación Biomédica en Red de Epidemiología y Salud Pública), Madrid, Spain; Mayo Clinic Rochester, UNITED STATES

## Abstract

**Background:**

Diagnostic delay is well-known in spondyloarthritis and studies have demonstrated a longer deferral in women. The aim of this study was to explore whether diagnostic delay of spondyloarthritis depends on clinical manifestations expressed by patients according to sex or whether it is related to a doctor’s misdiagnosis bias.

**Methods:**

A cross-sectional study of 96 men and 54 women with spondyloarthritis was performed at Alicante University General Hospital in Spain using a semistructured interview and clinical records. Comparative sex analysis were done via Student’s T and Mann-Whitney U tests for parametric and nonparametric continuous variables, chi-square and Fisher’s exact tests for unpaired categorical variables, and McNemar’s test for paired ones.

**Findings:**

The median diagnostic delay in women 7.5 (11.5) years is higher than men 4 (11) years, with a difference close to statistical significance (p = 0.053). A total of 30.2% of men received a first correct diagnosis of spondyloarthritis versus 11.1% of women (*p* = 0.016), indicating that men have higher probability of not having a misdiagnosis of spondyloarthritis (odds ratio = 3.5; 1.3–9). Eleven different health services referred male patients to the rheumatology clinic but only six in the case of female. No sex differences were detected in patients’ manifestations of back pain at onset. However, medical records registered differences (women 44.4%, men 82.1%; *p* < 0.001). There were differences between patients (women 57.7%, men 35.2%; p = 0.008) and medical records in peripheral signs/symptoms at onset (women 55.6%, men 17.9%; *p* < 0.001).

**Conclusion:**

The few differences of self-reported manifestations between both sexes with spondyloarthritis as compared with their clinical records (more axial pain in men and more peripheral pain in women) suggests that the professionals’ annotations reflect what they expect according to Literature, which could explain the higher misdiagnosis and diagnostic delay in women with spondyloarthritis.

## Introduction

Like myocardial infarction, spondyloarthritis has been considered a primarily male disease (i.e. a disease that has greater prevalence in men) and later thought of as a disease with sex differences in symptomatology. For decades, it has been underdiagnosed in women [1, 2]. Spondyloarthritis comprises a heterogeneous group of interrelated inflammatory rheumatic diseases that can affect the synovium, enthesis of the axial and peripheral joints, and certain extra-articular sites, such as the eye, skin, or gut [3].

Ankylosing spondylitis [4] (AS), described in the 19^th^ century draws the attention of physicians because of the dramatic spinal fusion that patients present. Around 1960, what were previously called reactive arthritis, enteropathic arthritis, juvenile spondyloarthritis, and some forms of psoriatic arthritis were identified as independent diseases. The epidemiological studies of Moll and Wright [3] showed familial aggregation of these diseases as a group (and occasional evolution of one to another), suggesting a close link among them. After describing the strong association between AS and HLA-B27 [5] in 1973, researchers found that other diseases were also associated with AS, confirming that these diseases were part of the same pathological process, now jointly referred as spondyloarthritis. AS was considered a rare disease in women [6, 7]. It was not until after 1975 that several authors began to realise that AS and spondyloarthritis were not as uncommon in women as previously believed [8, 9].

Former unawareness of a different presentation of spondyloarthritis in women as compared with men (i.e. less axial and more peripheral affection) is likely an important reason for the decreased diagnostic suspicion, increased misdiagnosis in women, and gender inequality in treatment approach and underestimation of its prevalence. Most studies of gender bias in the diagnostic effort are based on the unequal frequency by sex of objective tests requested for diagnosis. However, the diagnosis of spondyloarthritis is based, at onset, on medical record review and physical examination, where different (or even the same) symptoms and signs can result in different readings in either sex that ignite diagnostic suspicion depending on the knowledge and the perspectives of interpretation. This is where misdiagnosis bias may be most likely due to gender insensitivity [10].

The classification criteria of spondyloarthritis reflect the changes that have occurred in our understanding of spondyloarthritis through time. Rome’s diagnostic criteria of 1961 were the first. However, until 1990, with publication of the classification criteria of Amor and the European Spondyloarthritis International Society, peripheral damage was not included, which prevented the realization that the prevalence was not lower in women. In 2009, the Assessment of Spondyloarthritis International Group (ASAS) published differentiated classification criteria of axial and peripheral spondyloarthritis; the specific ASAS criteria for peripheral spondyloarthritis made the disease and its prevalence even more visible in women. Patients can now be classified as having axial spondyloarthritis, which includes AS and non-radiographic axial spondyloarthritis, and peripheral spondyloarthritis [11, 12]. However, although an increasing number of physicians now know that women can suffer from spondyloarthritis, many still link spondyloarthritis to the appearance and consultation of men, based on their professional imaginary and learned diagnostic practices.

Diagnostic delay is well-known in spondyloarthritis, and published studies have noted a longer deferral of diagnosis in women [1,2]. Patients have reported having to navigate for years a number of clinical pathways by different medical specialists and other care providers, including obtaining an alternative diagnosis, before spondyloarthritis is finally diagnosed [13,14]. Necessary and unnecessary tests are frequently performed based on patient symptoms. With recent conceptual advances in spondyloarthritis, and with an aim of avoiding structural damage, early diagnosis and awareness of sex differences has become possible and most desirable.

Most studies have simply measured the time delay between the onset of symptoms and start of treatment, without analysing where and why the delay occurs or whether the delay depends on the patient (patient delay) and/or the health professionals (medical delay). We performed a study in Spain using the universal healthcare system and three levels of medical care (primary, secondary with specialist non-rheumatologists, and the rheumatology clinic at hospital). The objective was to explore whether the diagnostic delay of spondyloarthritis depends on initial clinical manifestations expressed by patients according to sex, or whether it is related to the doctor’s misdiagnosis bias, more frequent in women, prior to spondyloarthritis diagnosis.

## Method

### Study design and participants

This study used a cross-sectional design to compared two groups of patients: 96 men and 54 women.

Inclusion criteria were patients >18 years old; diagnosed with spondyloarthritis that fulfilled modified New York criteria, European Spondyloarthropathy Study Group criteria, Amor criteria, or ASAS criteria; and who attended the Rheumatology Department of the University General Hospital of Alicante, Spain, during a 1-year period (March 2013–February 2014).

Exclusion criteria were patients with psoriatic arthritis, psoriasis, or inflammatory bowel disease. They were excluded because the published diagnostic delay is different and to avoid interference [15].

In total, five patients were excluded: three men and one woman due to the inability to recall information about their disease. Moreover, one woman did not provide consent due to a lack of time. The participation rate was 96.9% in men and 96.4% in women.

The ethics committee of the Alicante University General Hospital approved this study (Ref. CEIC PI2010/09). Before participating in the interviews, the subjects gave their verbal informed consent, and confidentiality of all information was guaranteed.

### Data sources

The sources of information were a semi-structured interview administered to patients (S1 Appendix) and review of clinical records to determine how rheumatologists registered the manifestations of patients.

From patient interviews, we obtained the following data: age, sex, family history of disease, smoking habit, body mass index, age at symptom onset (i.e. time when the first symptom, axial or peripheral pain, developed), and age at diagnosis. We asked for clinical data about back pain (lumbar, dorsal, cervical, costo-esternal, and buttock pain) and peripheral pain (shoulder, elbow, hands and wrist, hip, knee, ankle, heel, and feet pain) at onset of disease. Moreover, we recovered information about health pathways that patients followed before arriving at the rheumatology clinic (primary care and/or some specialists) and who provided the referral to the rheumatology clinic (primary care and/or some specialists and/or non-healthcare services). We also reviewed alternative diagnoses given to patients by general practitioners and other specialists before the diagnosis of spondyloarthritis. These diagnoses were classified as (1) misdiagnosis, (2) diagnosis related to signs and symptoms, and (3) diagnosis related to nonspecific causes.

The review of clinical records included the description provided by rheumatologists after conducting a physical examination. This included lumbar, dorsal, cervical, and costo-esternal pain; shoulder, elbow, hands, and hip arthritis; knee arthritis and enthesitis; and ankle and foot arthritis, dactylitis, and enthesitis. In addition, human leucocyte antigen (HLA-B27) status was acquired from records together with the definitive diagnosis (axial, peripheral, or mixed).

Disease duration (years) was calculated by deducting the date of the study interview from the date of disease onset. Diagnostic delay was considered the time difference (in years) between the onset of symptoms and the date of diagnosis by a professional.

### Statistical analysis

A descriptive analysis of the above-mentioned data was performed to compare sex-related differences in patients with spondyloarthritis, using means and 95% confidence intervals, medians and interquartile ranges for parametric and nonparametric continuous variables respectively, and frequencies and percentages for categorical variables. A comparative analysis was performed using the Student’s T-test for parametric continuous variables (current age, age at onset, age at diagnosis) and the Mann-Whitney U test for nonparametric (total diagnosis delay, patients´ delay and medical delay, disease duration and body mass index). The chi-square test –Pearson and linear trend—and the two-sided Fisher’s exact tests were used for unpaired categorical variables and the McNemar’s test for paired categorical ones. A p-value of <0.05 was considered statistically significant. To examine how sex is related to misdiagnosis, odds ratios (OR) with 95% confidence intervals were calculated. Statistical analyses were performed using IBM SPSS Statistics for Windows, (Version 21.0. Armonk, NY: IBM Corp.).

## Results

**Fig 1:**
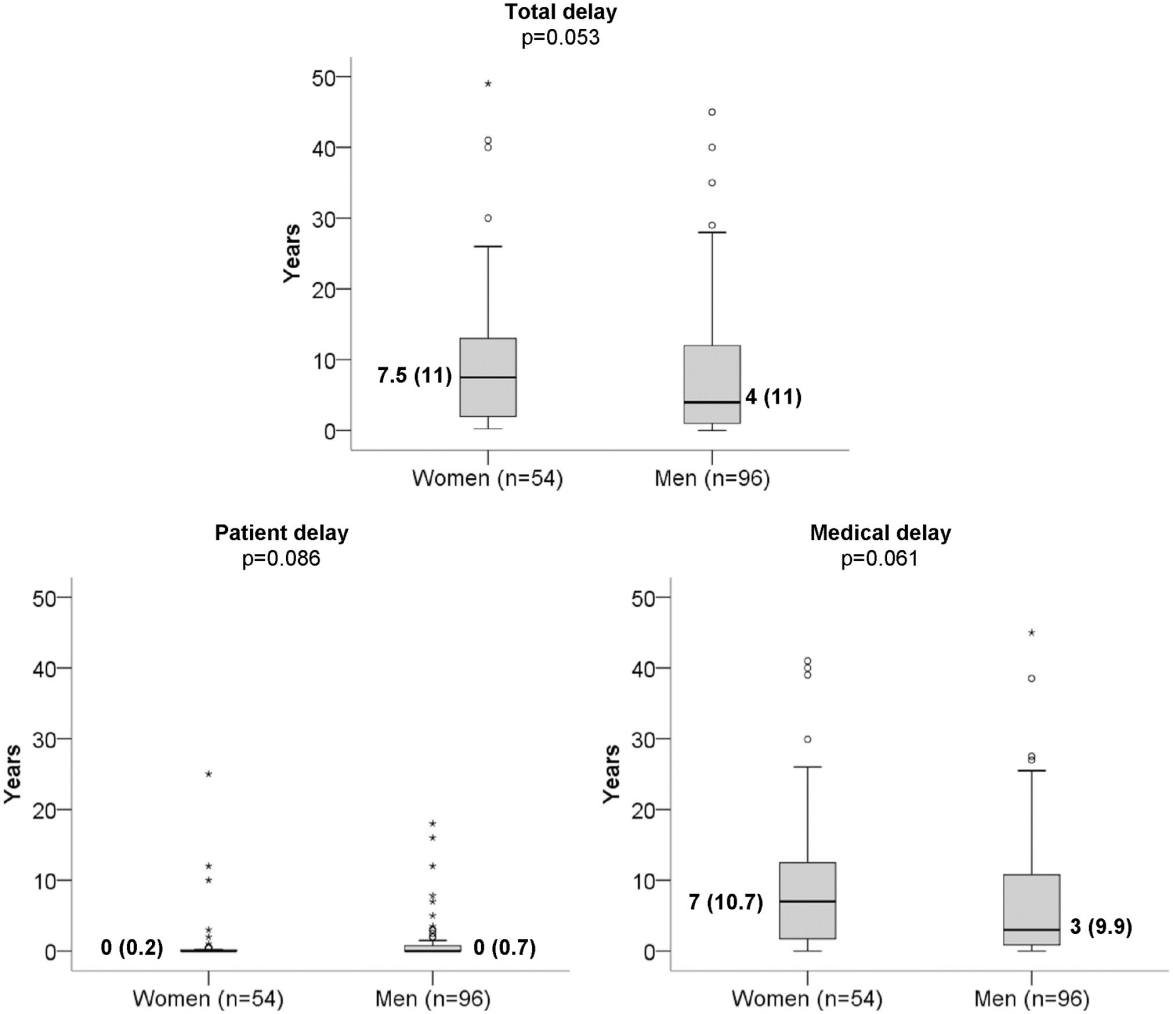
Differences by sex in the diagnostic delay of spondyloarthritis, total and according to medical and patient delay in the outpatient clinic of the General Hospital of Alicante (Spain, 2014). **Median and (interquartile range)**. Comparisons between men and women using the Mann-Whitney *U* test.

Fig 1 shows the pattern by sex of diagnostic delay of spondyloarthritis, with a median of 7.5 (11.5) years in women and 4 (11) years in men *p* = 0.053, which coincides much more with medical delay than with the delay of patients.

Diagnosis at the rheumatology clinics included axial spondyloarthritis (men: 45.8%, women: 25.9%), peripheral spondyloarthritis (men: 6.3%, women: 27.8%), and axial and peripheral spondyloarthritis (men: 47.9%, women: 46.3%; *p* = 0.0006) “Table 1”.

**Table 1:** Clinical characteristics of patients with spondyloarthritis in outpatient’s clinic of the General Hospital of Alicante (Spain 2014).

	Women (n = 54)	Men (n = 96)	*P* value
**Current age**			
Mean (95% CI), years	49.6 (45.8 to 53.4)	51.6 (48.9 to 54.3)	0.39^a^
**Age at onset**			
Mean (95% CI), years	31.2 (27.5 to 35.0)	30.9 (28.2 to 33.5)	0.87^a^
**Age at diagnosis**			
Mean (95% CI), years	41.9 (38.4 to 45.4)	39.1 (36.4 to 41.7)	0.20^a^
**Disease duration**			
Median (IQR), years	16.0 (16.0)	18.1 (21.5)	0.31^b^
**Type of Spondyloarthritis**			
Axial, n (%)	14 (25.9)	44 (45.8)	**0.0006** ^c^
Peripheral, n (%)	15 (27.8)	6 (6.3)	
Axial and peripheral, n (%)	25 (46.3)	46 (47.9)	
**Familiar history**			
Presence, n (%)	12 (22.2)	21 (21.8)	0.96^d^
**HLA-B27**			
Positive, n (%)	32 (59.3)	74 (77.1)	**0.02**^d^
**Tobacco**, n = 143			
Smoker, n (%)	10 (19.2)	25 (27.5)	0.27^d^
**Body Mass Index**, n = 137			
Median (IQR), kg/m^2^	25.1 (5.6)	25.9 (5.5)	0.41^b^

Data analysed in the variables: Tobacco, 52 women and 91 men smokers; Body Mass Index, 50 women and 87 men.

Values are mean and 95% confident interval (CI) for parametric continuous variables; median and interquartile range (IQR) for non-parametric continuous; number (n) and percentage (%) for qualitative.

^a^ Student’s T-test;

^b^ Mann Whitney U test;

^c^ Chi-square test for homogeneity;

^d^ Pearson’s Chi-square test.

Significant results are marked in bold (p < 0.05).

There were no detected differences between sexes for mean age (women: 49.6 (45.8–53.4) years, men: 51.60 (48.9–54.3) years), mean age at onset of disease (women: 31.2 (27.5–35.0) years, men 30.9 (28.2–33.5) years), mean age at diagnosis (women: 41.9 (38.4–45.4) years, men: 39.1 (36.4–41.7) years), or duration of disease (women: 18.4 (14.7–22.1) years, men: 20.7 (17.9–23.6) years). In addition, there was not detected difference between sexes in family history of spondyloarthritis (women 22.2%, men 21.8%). However, HLA-B27 positivity was present in 77.1% of men and in 59.3% of women (*p* = 0.02).

A total of 30.2% of men received a first correct diagnosis of spondyloarthritis versus 11.1% of women (*p* = 0.016). This means that men have a higher probability of not having a misdiagnosis of spondyloarthritis (OR = 3.5 (1.3–9)) “Table 2”.

**Table 2:** Number of erroneous diagnosis before the spondyloarthritis diagnosis in outpatient’s clinic of the General Hospital of Alicante (Spain 2014).

	Women (N = 54)	Men (N = 96)
First correct diagnosis	6 (11.1)	29 (30.2)
One previous diagnosis	23 (42.6)	35 (36.5)
Two previous diagnosis	15 (27.8)	16 (16.7)
Three previous diagnosis	5 (9.3)	4 (4.1)
Four previous diagnosis	2 (3.7)	0
Five previous diagnosis	1 (1.9)	3 (3.1)
Don’t know/ No answer	2 (3.7)	9 (9.4)

Values are number (percentage).

*P* value = **0.016** using Linear Trend Chi-square test.

“Table 3” presents the common diagnostic errors for both sexes and “Table 4” shows different misdiagnosis in women and men. Herniated disc was the most frequent alternative diagnosis to spondyloarthritis in men (19%) and the third most frequent in women (10.9%). Conversely, sciatica was the top misdiagnosis in women (19.6%) and the third most frequent in men (6.9%).

**Table 3:** Common misdiagnosis in both women and men before the spondyloarthritis diagnosis in outpatient’s clinic of the General Hospital of Alicante (Spain 2014).

	Women	Men
**Misdiagnosis**		
Herniated disc	5 (10.9)	11 (19.0)
Scoliosis	2 (4.4)	5 (8.6)
Sciatica	9 (19.6)	4 (6.9)
Osteoarthritis	6 (13.0)	3 (5.2)
Tendinitis	1 (2.2)	2 (3.5)
Fibromyalgia	3 (6.5)	1 (1.7)
Fasciitis	1 (2.2)	1 (1.7)
Gout	1 (2.2)	1 (1.7)
**Signs and symptoms**		
Low back pain	3 (6.5)	11 (18.97)
Psychosomatic pain	3 (6.5)	4 (6.90)
Cervical contracture	1 (2.2)	2 (3.45)
Arthralgia	2 (4.4)	1 (1.72)
**Non-specific causes**		
Rheumatism	2 (4.4)	6 (10.3)
Stress	3 (6.5)	1 (1.7)
For growth	2 (4.4)	1 (1.7)
**He/She has nothing**	3 (6.5)	2 (3.5)

Values are number (percentage)

**Table 4:** Different misdiagnosis in women and men before spondyloarthritis diagnosis in outpatient’s clinic of the General Hospital of Alicante (Spain 2014).

Only in women		Only in men	
**Misdiagnosis**			
Rheumatoid arthritis	2 (4.4)	Rheumatic fever	1 (1.7)
Bursitis	2 (4.4)	Spur	1 (1.7)
Osteoporosis	1 (2.2)	Sprain	1 (1.7)
Myofascial syndrome	1 (2.2)		
Conjunctivitis	1 (2.2)		
**Signs and symptoms**			
Lumbar contracture	2 (4.4)	Postural muscular pain	5 (8.5)
Bone sclerosis	1 (2.2)	Pinching	3 (5.2)
Vascular problem	1 (2.2)	Neuralgia	2 (3.5)
Lack of lumbar lordosis	1 (2.2)	Cervicalgia	1 (1.7)
One leg shorter than another	1 (2.2)	Muscle inflammation	1 (1.7)
		Sclerosis	1 (1.7)
		Myalgia	1 (1.7)
		Exhaustion	1 (1.7)
**Non-specific causes**			
For standing time	1 (2.2)	Suspected cancer	2 (3.5)
Obesity	1 (2.17)	By age	2 (3.45)
“Bone stuff”	1 (2.17)	Constipation	1 (1.72)
		“Maladjustment in Spain”	1 (1.72)
		“For playing football”	1 (1.72)
		“Too much car”	1 (1.72)

Values are number (percentage)

Before definitive diagnosis of spondyloarthritis, patients passed through different health care pathways “Table 5”. Most of the patients visited their primary care physician at least once (women: 81.5%, men: 89.6%). The average number of specialists visited was 1.2 (range, 1–1.4) in men and 1.61 (range, 1.3–2) in women (*p* = 0.05). Orthopaedic surgery evaluated 63% of women and 51% of men, followed by rehabilitation services, in which 16.7% of both sexes were attended. Sex differences were found in emergency department, with 38.9% of women and 15.6% of men attended (*p* = 0.001), and ophthalmology services, with 11.1% of women and 1% of men attended (*p* = 0.009). In addition, 14.6% men consulted to the workers’ compensation insurance versus 3.7% of women (*p* = 0.05).

**Table 5:** Health pathways of patients before the spondyloarthritis diagnosis in outpatient’s clinic of the General Hospital of Alicante (Spain 2014).

Health Services	Attending prior diagnosis in Rheumatology Clinic		*P* value	Referring to Rheumatology Clinic		*P* value
	Women (N = 54)	Men (N = 96)		Women (N = 54)	Men (N = 96)	
Primary care	44 (81.5)	86 (89.6)	0.16	14 (25.9)	30 (31.3)	0.49
Orthopedic surgery	34 (63.)	49 (51.)	0.16	11 (20.4)	23 (24.)	0.62
Rehabilitation services	9 (16.7)	16 (16.7)	1	0	2 (2.)	NA
**Emergency department**	**21 (38.9)**	**15 (15.6)**	**0.001**	**9 (16.7)**	**6 (6.3)**	**0.04**
Workers´ Compensation Insurance	2 (3.7)	14 (14.6)	0.05	**0**	4 (4.2)	**NA**
Neurology	2 (3.7)	4 (4.2)	1.	1 (1.9)	2 (2.1)	1.
Physiotherapy	2 (3.7)	3 (3.1)	1.	0	0	NA
Pain Unit	2 (3.7)	2 (2.1)	0.62	0	0	NA
Urology	1 (1.9)	2 (2.1)	1.	1 (1.9)	1 (1.)	1.
Gastroenterology	0	3 (3.1)	NA	0	2 (2.1)	NA
**Ophthalmology**	**6 (11.1)**	**1 (1.)**	**0.009**	**5 (9.3)**	**1 (1.)**	**0.02**
Rheumatology	3 (5.6)	1 (1.)	0.13	0	0	NA
Neurosurgery	1 (1.9)	1 (1.)	1.	0	0	NA
General internal medicine	0	1 (1.)	NA	0	1 (1.)	NA
Psychology	0	1 (1.)	NA	0	0	NA
Cardiology	0	1 (1.)	NA	0	2 (2.1)	NA
Vascular surgery	1 (1.9)	0	NA	0	0	NA
Gynecology	1 (1.9)	0	NA	0	0	NA
Intensive Care Unit	1 (1.9)	0	NA	0	0	NA
Non health Services ^a^	0	0	NA	11 (20.4)	14 (14.6)	0.36
Don’t know	0	0	NA	2 (3.7)	8 (8.3)	0.33

Values are number (percentage).

Statistical significance using Pearson Chi-square test or two-sided Fisher’s exact test for values < 5.

Significant results are marked in bold (p < 0.05).

^(a)^ Non health Services: Patient is a health professional (2 women, 1 man), Patient has a relationship with a health professional (6 women, 7 men), Suspicion due to relative with SpA (4 men), At the patient’s request (2 women, 2 men) or Podiatry’s advice (1 woman).

NA Non-applicable.

Table 5 shows that 77% of men and 76% of women were referred to rheumatology services by 11 different health services (primary and specialties cares and workers´ compensation insurance in the case of men and by only 6 from primary and specialties services in the case of women. Emergency department (16.7% of women, 6.3% of men; *p* = 0.04) and ophthalmology (9.3%, 1%; *p* = 0.02) referred to rheumatology more women than men “Table 5”. Also, some patients used other ways to arrive to rheumatology: 13 patients had a relationship with a health professional, 4 patients had self-suspicion due to having a relative with spondyloarthritis, 4 patients requested it, 3 patients were health professionals, and 1 patient arrived based on podiatry’s advice. This means that 20% of women and 14% of men reached rheumatology clinics indirectly through nonmedical referral.

Based on patient self-reports, there were no detected sex differences in the manifestations of back pain at the onset of spondyloarthritis “Table 6”. However, there were significant sex differences in early spondyloarthritis symptoms related to back pain according to the medical records, in which it is noteworthy that fewer women than men started with back pain (women: 44.4%, men: 82.1%; *p* < 0.001). Looking specifically at lumbar pain, there were no sex differences based on patient self-reports, but in the medical records, men appeared to debut significantly more often with lumbar pain than women (women: 40.7%, men: 71.6%; *p* < 0.001). Therefore, medical records registered less back pain in women (44.4%) than those related by patients (61.5%) (p = 0.02). Similarly, medical records registered more lumbar pain in men as compared with patient reports (52.1% reported by men, 71.6% reflected in records; *p* = 0.003).

**Table 6:** Manifestations at onset. Back and peripheral symptoms and signs of patients with SpA in outpatient’s clinic of the General Hospital of Alicante (Spain 2014).

Affectation	According with patients			According with medical records			Patients vs Records	
	Women (n = 52)^a^	Men (n = 94)^b^	*P* value*	Women (n = 54)	Men (n = 95)^c^	*P* value*	In women *P* value**	In men *P* value**
**Back pain**	32 (61.5)	70 (74.5)	0.10	24 (44.4)	78 (82.1)	**<0.001**	**0.02**	0.19
Lumbar	24 (46.2)	49 (52.1)	0.49	22 (40.7)	68 (71.6)	**<0.001**	0.63	**0.003**
Dorsal	2 (3.9)	5 (5.3)	1.00	2 (3.7)	5 (5.3)	1.00	1.00	1.00
Cervical	1 (1.9)	10 (10.6)	0.10	2 (3.7)	7 (7.4)	0.49	1.00	0.38
Costo-esternal	0	6 (6.4)	NA	0	1 (1.1)	NA	NA	0.22
Buttock	7 (13.5)	7 (7.5)	0.24	0	0	NA	NA	NA
**Peripheral**	30 (57.7)	33 (35.2)	**0.008**	30 (55.6)	17 (17.9)	**<0.001**	0.82	**<0.001**
Hands/ wrist	8 (15.4)	3 (3.2)	**0.02**	6 (11.1)	2 (2.1)	**0.03**	0.69	1.00
Ankles/ feet/ heel	17 (32.7)	15 (16.)	**0.02**	20 (37.)	11 (11.6)	**<0.001**	0.63	0.63
Hip	3 (5.8)	8 (8.5)	0.75	0	0	NA	NA	NA
Knee	5 (9.6)	7 (7.5)	0.65	6 (11.1)	3 (3.2)	0.07	1.	0.38
Shoulder	4 (7.7)	3 (3.2)	0.25	0	0	NA	NA	NA
Elbow	0	0	NA	0	1 (1.)	NA	NA	NA

Values are number (percentage).

Missing data:

^a^ One woman expressed non-specific arthritis at onset and another woman uveitis, both of them were not included;

^b^ Two men expressed non-specific arthritis, non-included;

^c^ One man showed uveitis according to medical records, non-included.

* Comparison of independent samples using Pearson’s Chi-square test or two-sided Fisher’s exact test for values below five.

** Comparison between patients and medical records for women and for men using McNemar´s test for paired samples.

Significant results are marked in bold (p < 0.05).

NA Non-applicable.

Peripheral symptoms were significantly more frequent in women, both by self-report (women 57.7%, men: 35.2%; *p* = 0.008), and registered in the medical records (women: 55.6%, men: 17.9%; *p* < 0.001), as can be seen in Table 6. However, medical records documented less peripheral pain than men reported (*p* < 0.001).

## Discussion

Our findings confirm the existence of gender bias in the medical care of spondyloarthritis, defined as the differential medical treatment of men and women, the impact of which may be positive, negative or neutral [16]. The case of spondyloarthritis seems to be another case of Yentl syndrome. This syndrome was described, in the case of heart attacks, as the different medical care process usually followed according to the sex of the patients, and related to misdiagnosis in women because their symptoms/signs can differ from male symptoms. It highlights that unless women show the same male symptoms/signs; they will not be admitted to specialized attention and will receive the appropriate diagnostic and treatment [17].

In our case, in the timeline between the onset of spondyloarthritis symptoms and its diagnosis in the rheumatology clinic, female patients were misdiagnosed more times and experienced a longer delay in reaching a final diagnosis of spondyloarthritis as compared with male patients. The recording rheumatologists seem to have selected for annotation the symptoms referred by the patients that better fulfilled their preconceptions about spondyloarthritis in men and women, that is, back pain in men, more peripheral pain in women. This selection shows how the physician’s mental scheme weighs on the recorded notes and consequent diagnostic process, which may be hampered when relevant manifestations are left aside. Gender inequalities in spondyloarthritis may be understood as an indicator (and outcome) of gender inequity in the implementation of clinical practices, which is avoidable with analysis of sex and gender interactions that point out how gender bias in medical care remains.

Patients of both sexes sought medical attention for pain. Although the delay in diagnosis in recent years has been reduced [18–20], we observed an important diagnosis delay in both sexes, which was significantly higher in women. Despite the fact that men and women waited similarly from the beginning of symptoms to their first visit to a physician, after that, women had to wait longer than men until receiving a definitive diagnosis of spondyloarthritis. Therefore, if both sexes sought medical attention at same time, sex differences in diagnosis delay are mainly the fault of the health system [2], and this phenomenon has typically been explained as a lower level of clinical suspicion based on sex differences in clinical manifestations [14].

It is noteworthy that on the first clinic visit, about one-third of men received a correct diagnosis whereas only one-tenth of women did. According to patients, there were no sex differences in back pain at the beginning of the disease, although a higher proportion of women recalled peripheral symptoms. The difference in early diagnosis found between both sexes likely relates to the differential diagnosis scheme that physicians had in relation to the presenting symptoms and from a general medical perspective, because until recently, spondyloarthritis was labelled as ankylosing spondylitis and taken to be a predominately male and spinal disease [14]. This may be the reason why, upon the visit to the first physician, many male patients presenting with low back pain were recognized as having spondyloarthritis, whereas in women, other possibilities were considered first or spondyloarthritis was simply not considered. Those patients in whom spondyloarthritis was not initially recognized (more women than men) followed pathways of greater complexity and received a larger number of misdiagnoses before a final diagnosis of spondyloarthritis, likely explaining the longer diagnostic delay in women. The most frequent misdiagnoses have been described as a cause of diagnosis delay in spondyloarthritis [20, 21].

An important question from the viewpoint of gender bias is whether, when confronted with a similar clinical presentation in men or women, the physician considers different diagnostic alternatives, that is, has a different differential diagnosis in each sex. Our data show that sciatica and fibromyalgia were diagnosed in more women than men. Conversely, herniated disc was diagnosed in more men than women, and rheumatoid arthritis and bursitis were considered only in women. These data are agreement with the Literature, which has found a higher prevalence of fibromyalgia and rheumatoid arthritis in women [22, 23] as well as an increased expectation of herniated disc pathology in men because of increased prevalence in this population [24]. Symptoms similar to those of enthesitic pain can be observed in overweight people, individuals with fibromyalgia, and people who perform sports [20], and they can be misunderstood, as in our sample. Inflammatory back pain is not detected before arriving to rheumatology clinic and this may be at the base of misdiagnosis. The volume and diversity of unspecific diagnostics, such as cervicalgia, postural pain, lumbar contracture, or stress, reflect the uncertainties felt by the diagnosing physician when confronting these patients.

Most patients of both sexes were attended in primary care, which refers more patients to rheumatology (where spondyloarthritis is diagnosed), but only one-third of male and one-fourth of female patients were derived from the primary level. Altogether, men were referred to specialists more often than women. This may be one of the main reasons why women seek medical attention in the Hospital Emergency Department more often than men, from where they are subsequently referred to the rheumatology clinic. In addition, some patients used other ways to arrive at the rheumatology clinic, such as through nonmedical referral, which shows that primary and secondary health levels do not offer an adequate response to these patients.

We observed fewer differences between symptoms reported by women and men who had finally arrived at the rheumatology clinic as compared with those registered in the medical records of these patients. The recording rheumatologists seem to have paid more attention to the symptoms that they would expect to occur (i.e. back pain in men, peripheral pain in women), and their annotations seem to reflect their expectations. The rheumatologists may have written only those symptoms referred by the patients that better fulfilled their patterns, showing how the mental scheme of a physician weighs in the recorded notes and how it may influence the diagnostic process by leaving unconsidered manifestations of high diagnostic relevance. Medical records do not reflect exactly what patients refer and this could affect prognostic, treatment and wellness of patients.

The medical record is a tool used by physicians to register the history of symptoms and signs reported by patients that the physicians consider relevant, and it is one of the most important sources of information for diagnosis. Both medical record and the story of the patient are valuable, whether they coincide or not, as is evidence in stories about medically unexplained disorders, where the physician and patient are often at odds on the core issue of trust [25]. Diagnosis depends on the patient’s ability to describe their symptoms and on the doctor’s knowledge and clinical skills to interpret and observe the patient, which are sometimes partial. This seems to have occurred in our study.

Our study presents some limitations. As in other studies of clinical epidemiology in spondyloarthritis [2], with the exception of studies made from registers, the simple size is small. It may have limited the statistical power to detect differences according to the sex of patients in the variables under study, such as the delay in medical diagnosis and to detect differences between the self-report of symptoms of women with spondyloarthritis and data collected in clinical records. However, the study represents the spondyloarthritis population by sex and coincides in the frequency of clinical manifestations with the National Registry of Spondyloarthritis Patients in Spain [26].

The design was cross-sectional, making the direction of gender effects unclear in spondyloarthritis medical care; there might be sex differences in the experience of medical care that could be attributed to social factors other than gender that we do not collect. Although participants presented a wide range of conditions, severity degrees, illness durations, treatments, and ages, our results may be suitable for extrapolation to sociocultural contexts and health services similar to Spain. Patient interviews may have recall bias because of the long delay in diagnosis. However, we were confident about minimizing them because patients interviewed attended the rheumatology clinic at hospital, and this likely has a positive influence on the findings in terms of reducing recall bias.

In conclusion, taking our results with care due to their sample size, these seem to indicate that the large pathway of visits to different physicians and health professionals and the variety of diagnoses given to the patients in our study outline the difficulties of an early diagnosis of spondyloarthritis, which is important for initiating adequate treatment to avoid the progression of the disease and improve patient discomfort and the functional capacity. In addition, early diagnosis can help to avoid the pilgrimage of patients through different pathways in the health system, to decrease the anxiety of patients, and to save resources in terms of clinic visits and diagnostic tests. However, axial and peripheral spondyloarthritis seem to be an unknown disease, mainly in women. In the model of healthcare such as is implemented in Spain, the three levels of medical care present challenges for the diagnosis of spondyloarthritis. Different primary care approaches for shortening the time to diagnosis have been proposed. The RADAR study [27] and Esperanza healthcare programme [28] were useful for decreasing the diagnostic delay in spondyloarthritis. Further research should be conducted to show whether the prevalence of spondyloarthritis is underestimated in women [29], and, if so, whether the manifestations of spondyloarthritis are different by sex or whether there is bias in the registration of information. In addition, future studies should determine whether new knowledge about spondyloarthritis attained in recent years is being applied with gender equity at the different health care levels.

## Supporting information

S1 Appendix Questionnaire to patients. (DOCX)
