# Supplementary material for: Challenges to conquer from the gender perspective in medicine: The case of spondyloarthritis
Source: PLoS One. 2018 Oct 12;13(10):e0205751. doi: 10.1371/journal.pone.0205751 (PMC6185841; doi:10.1371/journal.pone.0205751)
Supplement: S1 Appendix — (DOCX) [file pone.0205751.s001.docx]

**Questionnaire to patients (English version)**

1. Can you tell which year or at what age began your Spondyloarthritis?
2. When was the Spondyloarthritis diagnosed?
3. Do you remember how long did you take from the start of the first symptom until you sought medical attention?
4. How long was since you requested medical attention until you were attended?
5. Which was your first symptom, complain, problem?
6. When you had the first symptom, did you go to a primary doctor, a specialist doctor, public, private?
7. How many doctors did you see before diagnosis of Spondyloarthritis?
8. Who referred you to Rheumatology?
9. Which alternative diagnosis did you receive before being diagnosed with Spondyloarthritis?
10. Are you a smoker? If not, have you ever smoke?
11. Which is your weight and height?
12. Do you have relatives with any rheumatic disease? If yes, which one?

**Cuestionario a pacientes (Versión española)**

1. ¿Podría decir qué año o a qué edad cree que comenzó la espondiloartritis?
2. ¿Cuándo le fue diagnosticada la EA?
3. ¿Recuerda cuanto tardó desde que comenzó el primer síntoma hasta que buscó atención médica?
4. ¿Cuánto tiempo transcurrió desde que solicitó atención médica hasta que fue atendido?
5. ¿Cuál fue su primer síntoma, queja, problema?
6. Ante el primer síntoma, ¿acudió a un médico de primaria, a un médico especialista, público, privado?
7. ¿Cuantos médicos visitó antes del diagnóstico de espondiloartritis?
8. ¿Quién lo derivó a Reumatología?
9. ¿Qué diagnósticos alternativos recibió antes del diagnóstico de Espondiloartritis?
10. ¿Es usted fumador? Si no lo es, ¿ha fumado alguna vez?
11. ¿Cuál es su peso y altura?
12. ¿Tiene familiares con enfermedades reumáticas? ¿Cuales?
